# Serum-Free Suspension Culture of the *Aedes albopictus* C6/36 Cell Line for Chimeric Orthoflavivirus Vaccine Production

**DOI:** 10.3390/v17020250

**Published:** 2025-02-12

**Authors:** Joshua S. Dawurung, Jessica J. Harrison, Naphak Modhiran, Roy A. Hall, Jody Hobson-Peters, Henry de Malmanche

**Affiliations:** 1 School of Chemistry and Molecular Biosciences, The University of Queensland, St Lucia, QLD 4072, Australia; j.dawurung@student.uq.edu.au (J.S.D.); j.harrison1@uq.edu.au (J.J.H.); n.modhiran@uq.edu.au (N.M.); roy.hall@uq.edu.au (R.A.H.); j.peters2@uq.edu.au (J.H.-P.); 2 Australian Infectious Diseases Research Centre, The University of Queensland, St Lucia, QLD 4072, Australia

**Keywords:** suspension culture, C6/36, vaccines, orthoflavivirus chimaeras, virus bioprocessing

## Abstract

Chimeric orthoflaviviruses derived from the insect-specific Binjari virus (BinJV) offer a promising basis for safe orthoflavivirus vaccines. However, these vaccines have so far only been produced using adherent C6/36 *Aedes albopictus* mosquito cell cultures grown in serum-supplemented media, limiting their scalable manufacture. To address this, we adapted C6/36 cells for serum-free suspension culture using Sf900-III medium, achieving high peak cell densities (up to 2.5 × 10^7^ cells/mL). Higher agitation rates reduced cell aggregation, and cryopreservation and direct-to-suspension revival were successful, confirming the adapted line’s stability for research and industrial applications. Despite this, BinJV-based chimeric orthoflaviviruses, including BinJV/WNV_KUN_, a candidate vaccine for West Nile virus, and similar vaccines (BinJV/DENV2 and BinJV/JEV_NSW22_) for dengue 2 virus and Japanese encephalitis virus, respectively, exhibited substantially reduced titres in C6/36 cultures infected in Sf900-III, a phenomenon attributed to the medium’s acidic pH. Switching to the more alkaline, serum-free CD-FortiCHO medium enhanced the replication of these chimeric viruses to peak titres between 1.7 × 10^7^ and 7.6 × 10^9^ infectious units per mL whilst preserving viral integrity. These findings suggest that suspension-adapted C6/36 cultures in CD-FortiCHO medium can support high-yield vaccine production for various orthoflaviviruses and highlight the important role of cell culture media pH for orthoflavivirus bioprocessing. This scalable mosquito cell-based system could reduce production costs and improve vaccine accessibility, supporting efforts to combat arbovirus-related public health challenges.

## 1. Introduction

The origin of the C6/36 cell line (*Aedes albopictus*; ATCC CRL:1660) dates back to 1967 when a polyclonal cell line was derived from newly hatched *Aedes albopictus* larvae [1]. This line was then cloned by rounds of limit dilution with the resultant C6/36 subclone being selected based on its ability to replicate arthropod-borne viruses (arboviruses) to significantly higher titres than other mosquito lines [2]. Indeed, the C6/36 cell line supports the replication of many arboviruses and serves as a widely used and valuable tool for detecting, amplifying, and analysing mosquito-borne viruses. Notably, the C6/36 cell line is deficient in a pivotal antiviral defence mechanism, the RNA interference (RNAi) pathway, rendering cultures exceptionally susceptible to arbovirus infection and capable of yielding substantial titres of infectious virus [3,4]. C6/36 cells are also noted for their diploid chromosome count (2n = 6) and non-anchorage-dependent and non-tumorigenic properties [5]. Because of these properties, the C6/36 cell line shows significant potential as a cell substrate for the replication of wild-type and chimeric viruses to mass-produce vaccines and diagnostic antigens. Whilst mammalian cell cultures are currently used to produce live chimeric, live attenuated, and inactivated arbovirus vaccines in Vero cells [6], newly developed arbovirus vaccine platforms based on chimeric mosquito-specific viruses [7,8] can only be replicated in mosquito cell substrates, necessitating the use of a cell line such as C6/36 [9]. For research purposes, C6/36 cultures are conventionally cultivated as adherent cultures in minimal media supplemented with animal serum-derived components, typically foetal bovine serum (FBS). This practice of using adherent cultures has posed challenges for large-scale bioprocessing due to its inherent limitations in scalability and the associated high costs [10,11,12]. Furthermore, the utilisation of culture systems containing animal serum supplements is unsuitable for the large-scale production of vaccines, due to the inconsistency in composition between different serum batches, the elevated protein content that complicates product purification, and the risk of contamination by adventitious viral, mycoplasma, or prion agents [13]. In this context, the utilisation of suspension cultures in serum-free media emerges as a more viable option. This alternative allows for higher cell densities (as cell proliferation is proportional to volume rather than attachment surface area) and the potential for increased virus yields per litre of culture medium, facilitating more efficient scaling of upstream culture systems and simplifying downstream purification processes [14].

The growth of C6/36 cells in suspension, and their use for viral culture, has been documented in only a few studies [15,16,17]. Morita and Igarashi [17] conducted a brief assessment of true suspension cultures in medium supplemented with FBS, achieving densities of up to 10^6^ cells/mL; however, they transitioned to using microcarriers for culturing Japanese encephalitis virus (JEV), maintaining a technically anchorage-dependent approach. Suchman and Carlson [16] successfully adapted C6/36 cells to suspension culture using the Sf900-II serum-free medium to cultivate densovirus in this system. By shaking cultures at approximately 280 rpm, cell clumping was eliminated, and a cell density of 3 × 10^7^ cells/mL was achieved; nonetheless, the method was not tested with arboviruses. This study was extended by Grzenia et al. [15] with *Aedes aegypti* densovirus-infected C6/36 cultures scaled to 0.5 L bioreactors, although the culture of arboviruses was again not explored.

We recently developed a novel chimeric virus system using a newly discovered insect-specific orthoflavivirus, Binjari virus (BinJV), to produce vaccine candidates [18]. By swapping the prM/E structural genes of BinJV with those of pathogenic orthoflaviviruses, we created chimeric virions that mimic the structure of the pathogenic viruses and accurately present potent, neutralising, antibody-eliciting quaternary epitopes, essential for robust immune responses. These chimeric viruses replicate to high titres in mosquito cell cultures, but retain the insect-specific phenotype of BinJV, making them incapable of replicating in vertebrate cells and ensuring they cannot cause disease in humans or other vertebrates [7]. This combination of safety, efficacy, and ease of production makes these chimeric viruses excellent alternative vaccine candidates, offering a promising strategy for preventing orthoflavivirus infections [17,18,19,20].

The development of commercial orthoflavivirus vaccines based on chimeric mosquito-specific backbones must overcome challenges such as non-scalable static cultures, a reliance on ultracentrifugation for particle purification, the use of animal products in media, incompatibilities with current good manufacturing practice (cGMP), and uncertainties regarding the regulatory acceptance of the novel insect/mosquito virus–cell system. To address these issues, it is crucial to develop a safe and efficient vaccine production bioprocess to facilitate industrial scale-up and ensure compliance with international regulatory standards. In this study, we established a suspension culture system for C6/36 cells using commercially available serum-free medium devoid of proteins and animal-derived components. Moreover, we demonstrated the practical application of this system by assessing its capacity to replicate chimeric BinJV-based vaccine candidates against West Nile virus, Japanese encephalitis, and dengue fever.

## 2. Materials and Methods

### 2.1. Cell Line, Culture Media, and Viruses

Adherent C6/36 (*Aedes albopictus*) cells of undocumented passage history were obtained from laboratory stocks and grown and maintained in RPMI 1640 (Gibco, Grand Island, NY, USA) supplemented with 2.5% FBS, 50 U/mL penicillin, 50 μg/mL streptomycin, and 2 mmol/L L-glutamine and incubated at 28 °C. Alternatively, C6/36 cultures were maintained in Sf900-III (Gibco, Grand Island, NY, USA), a serum-free medium (SFM) used for insect cell cultures that is devoid of animal-origin components, or CD-FortiCHO (Gibco, Grand Island, NY, USA), a chemically defined mammalian cell culture media. The chimeric viruses used in all the infection experiments (BinJV/WNV_KUN_, BinJV/JEV_NSW22_, and BinJV/DENV2) have been previously described [17,19,21,22].

### 2.2. Adaptation and Maintenance of C6/36 Suspension Cultures

The transition of adherent C6/36 cells from an FBS-supplemented culture to a serum-free condition was accomplished through a series of steps. Initially, FBS-supplemented adherent C6/36 cells cultured in RPMI 1640 medium (Gibco, Grand Island, NY, USA) were trypsinized and seeded in 6-well plates (ThermoFisher, Waltham, MA, USA) with a working volume of 2 mL per well (0.5 mL of cells in 1.5 mL of medium). Following cell attachment, the medium was removed and replaced entirely with 2 mL of Sf900-III (Gibco, Grand Island, NY, USA). The cells were closely monitored daily until 100% confluency was reached. At this point, the cells were gently detached from the plate surface by pipetting and then split at a ratio of 1:3 into multiple new wells (0.5 mL of cell suspension and 1.5 mL of fresh Sf900-III per well). Subsequently, the cells were monitored daily until 100% confluency was achieved again. Once this was attained, the cells from each well were gently dislodged and pooled (12 mL total) for adaptation to suspension culture in Sf900-III, within a 125 mL vented Erlenmeyer flask in a total culture volume of 25 mL. The flask was then incubated at 28 °C on an orbital shaker (25 mm orbital diameter) operating at 250 rpm. Passage events occurred when the cells reached a cell count of 2–2.8 × 10^7^ cells/mL, occurring roughly every 14 days following initial adaptation, and were then typically conducted once per week once cultures were fully adapted and cryopreserved. The cultures were typically split at a ratio of 1:4 targeting a seeding density of 5 × 10^6^ cells/mL. Daily assessments of cell counts and viability were conducted using a hemocytometer.

### 2.3. Cell Growth, Viability, and Doubling Time

Cell growth and viability were determined in the presence of 0.05% Trypan Blue (Invitrogen, Grand Island, NY, USA) by counting the total number of cells and viable cells in a Neubauer chamber hemocytometer (Boeco, Hamburg, Germany) under inverted light microscopy. The population doubling time (DT) was estimated using the equation below:

DT = (t × ln 2)/(ln N − ln N_0_)

where t is the cultivation time in hours, N is the final cell density, and N_0_ is the initial cell density.

### 2.4. Cryopreservation and Revival of C6/36 Suspension Cells in Serum-Free Media

Solution containing Sf900-III supplemented with 20% cell culture-grade dimethyl sulfoxide (DMSO), termed freeze medium, was prepared and cooled to 4 °C. A volume of cell suspension harvested from mid-exponential cultures was centrifuged at 200× *g* for 5 min, and the cell pellet was resuspended in the chilled freeze medium to reach a cell density of 2 × 10^8^ cells/mL. The resuspended cells were further diluted in an equal volume of chilled conditioned Sf900-III medium so that the final concentration of DMSO was 10% and the cell density was 1 × 10^8^ cells/mL. The suspension was aliquoted in a final freeze volume of 1.8 mL and dispensed into 2.0 mL cryotubes. These vials were loaded into a cryopreservation device (Mr. Frosty^TM^ Freezing Container; Nalgene, Monterrey, Mexico) and stored overnight at −80 °C prior to transfer to liquid nitrogen for long-term cryopreservation. To revive the cells, the cryovial was removed from liquid nitrogen and rapidly thawed to room temperature. The contents were mixed with 10 mL of chilled culture medium (Sf900-III) then centrifuged at 200× *g* for 10 min to remove the DMSO. After centrifugation, the cell pellets were resuspended in fresh Sf900-III to 25 mL and transferred into a 125 mL vented Erlenmeyer flask for direct revival in shaking culture.

### 2.5. Virus Infection and Harvesting in Adherent Culture

C6/36 cells were initially cultured to attain 80% confluency in RPMI 1640 medium (Gibco) supplemented with 2% FBS and supplemented with penicillin (50 U/mL), streptomycin (50 µg/mL), and 2 mM L-glutamine (PSG). Subsequently, these cells were infected with BinJV/WNV_KUN_ at a multiplicity of infection (MOI) of 0.1. The infected cultures were then incubated at a temperature of 28 °C for a duration of 7 days. Following this incubation, the virus-containing supernatant was harvested by centrifugation at 1700× *g* for 30 min, and the resulting supernatant stored at a −80 °C.

### 2.6. Quantification of Infectious Viral Titre

The infectious viral titre was determined by the titration of the viral supernatant in C6/36 cells and expressed as infectious units (IU)/mL and performed according to previously published methods [7]. Briefly, C6/36 cells were seeded in 96-well plates to achieve around 80% confluency and were left to incubate overnight. Then, 50 µL of serially, ten-fold diluted virus (8 replicates per dilution) was added to each well, and the cells were further incubated for 5 days. After incubation, cell monolayers were fixed overnight at 4 °C in PBS with 20% acetone. Infected wells were identified by probing the plates with the pan-orthoflavivirus NS1-reactive murine mAb 4G4 [23] followed by the horseradish-conjugated goat anti-mouse antibody, each for 1 h at 37 °C. Between the primary and secondary antibody steps, the plates were washed with PBS containing 0.5% Tween-20. ABTS substrate solution was added and allowed to incubate for 1 h in the dark; then, optical density was measured using an automated plate reader set at 405 nm. Positive wells were scored with reference to the negative controls (uninoculated wells) and the TCID_50_ was calculated [24].

### 2.7. Virus Purification

The purification was performed as described previously [7]. The virus-containing culture supernatant was clarified by centrifugation at 1700× *g* for 30 min, followed by filter-sterilisation (0.20 µm). Polyethylene glycol 8000 (PEG) was added to the virus in the clarified culture supernatant to give a final PEG concentration of 8%. Precipitation was performed by the gentle vortexing of the mixture at 4 °C overnight. Subsequently, centrifugation was carried out at 4 °C at 11,900× *g* for one hour using an Avanti J-26 JLA10.5 rotor. The virus pellet was then resuspended in NTE buffer composed of 12 mM Tris, 120 mM NaCl, and 1 mM EDTA (pH of 8), followed by ultra-centrifugation through a 20% sucrose cushion at 133,907× *g* (SW32Ti rotor) for 2 h at 4 °C. The resulting pellet was re-suspended in 500 µL of NTE buffer and kept at 4 °C overnight. The resuspended virus solution was then clarified at 100 g for 10 min; then, the virus was purified through a 25–40% potassium tartrate gradient and centrifuged at 336,840× *g* at 4 °C for 1 h (SW60 rotor). The visible bands were collected from the tube and subjected to buffer exchange into cold sterile PBS using a 30 kDa Amicon filter (Millipore, Burlington, MA, USA). The purified virus was stored at 4 °C until further use.

### 2.8. Infection of C6/36 Cells in Suspension

Cell density was determined through cell counting, and cells were diluted in fresh medium and seeded in Erlenmeyer flasks to give approximately 5 × 10^6^ cells per mL. The split ratio indicated that for every 1 part of cells in Sf900-III medium, there should have been 4 parts of either Sf900-III medium or CD-FortiCHO (Gibco) medium (the latter utilising a medium-swap technique). These cultures were then subjected to infection with a virus (BinJV/WNV_KUN,_ BinJV/JEV_NSW22_, or BinJV/DEN2) at a multiplicity of infection (MOI) of 0.1. Following infection, the cultures were incubated at a temperature of 28 °C at 250 rpm for a period of 7 days. At various times post-infection, the supernatant from three biological replicate-infected cultures was harvested, clarified by low-speed centrifugation, and preserved at −80 °C for subsequent analysis.

### 2.9. Immunofluorescence Assay (IFA)

To prepare cells for IFA, inoculated C6/36 monolayers grown on glass coverslips were fixed by being submerged in 100% ice-cold acetone and dried at room temperature. IFA analysis was subsequently conducted following the detailed procedures outlined in previous protocols. These assays utilised the mouse pan-orthoflavivirus reactive NS1 monoclonal antibody (mAb) 4G4 [24]. This specific antibody targets the NS1 protein, a common antigenic component across various orthoflaviviruses known to bind to the BinJV NS1 protein. Subsequently, the coverslips were mounted onto slides using Prolong Gold glue for imaging. Images were captured using a ZOE^TM^ Fluorescent cell imager (BIO-RAD, Singapore).

### 2.10. Negative Staining Transmission Electron Microscopy (TEM) of Purified Virus Preparations

The culture supernatants were harvested and the viruses were purified through potassium tartrate gradients as described above. The purified virus particles were diluted to approximately 400 µg/mL in PBS and 4 μL was adsorbed onto carbon-coated grids (ProSciTech, Thuringowa, QLD, Australia) for 2 min and glow-discharged for 5 s at 25 mA. The grids underwent blotting and were washed three times in water, followed by staining twice with 2% uranyl acetate with blotting in between. After air-drying, the grids were imaged using a Hitachi HT7700 microscope (Hitachi High-Technologies, Tokyo, Japan) operated at 120 Kv.

## 3. Results

**Figure 1:**
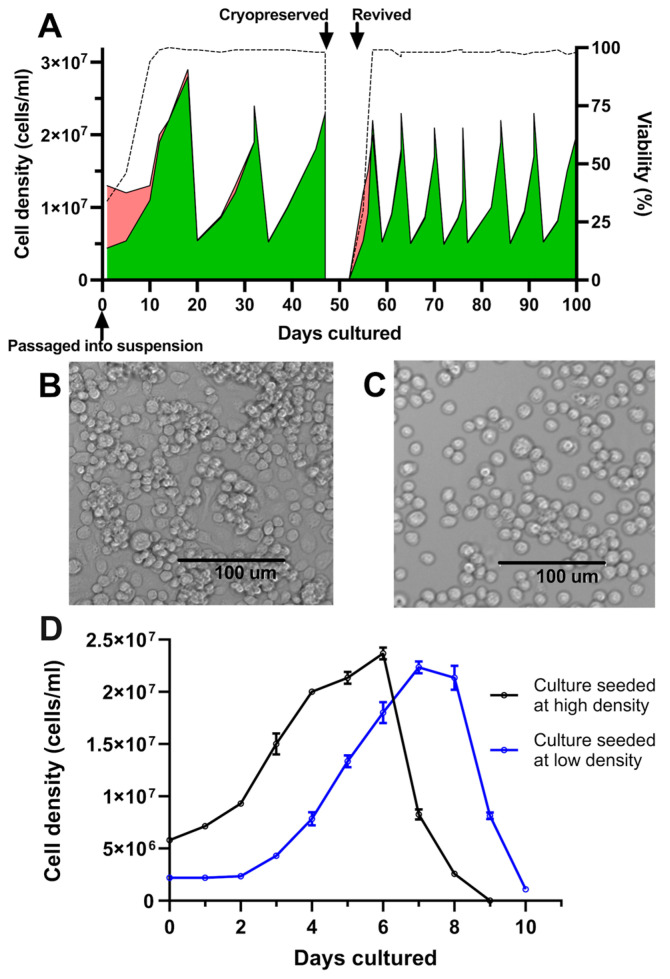
Growth kinetics and morphology of C6/36 cultures following adaptation to suspension. (**A**) Cell count and viability of 25 mL C6/36 suspension cultures following transition into shake flasks and continual passage via dilution into fresh media, tracking culture behaviour following cryopreservation and revival. Green areas represent live cells; red areas represent dead cells; and dotted line represents culture viability. (**B**) Culture morphology at low shake speed (120 rpm). (**C**) Culture morphology at high shake speed (250 rpm). (**D**) C6/36 suspension cultures were seeded in triplicate at two different cell densities and viable cell density was enumerated daily. Mean viable cell density is plotted with error bars representing +/− one standard deviation.

Following the initial transition from adherent to suspension culture in Sf900-III in shake flasks, C6/36 cultures exhibited an initial period of poor growth, marked by a temporary decline in cell viability (Figure 1A). Subsequently, the cells demonstrated consistent growth and achieved an average of 98% cell viability. Following three passages over a period of 47 days, ampoules of the culture were successfully cryopreserved and subsequently revived directly into suspension, underscoring the adaptation of these cells to suspension culture in serum-free media (Figure 1A). During initial experiments, cultures underwent testing at two shake speeds, 120 and 250 rpm. At 120 rpm, a shake speed typically used for insect suspension cultures, undesirable cell clumping occurred (Figure 1B). However, clumping was not observed after increasing the speed to 250 rpm (Figure 1C). Morphological assessment revealed a singular, rounded, and compact appearance of the cells.

After adaption to suspension culture, passaging parameters were determined empirically by assessing two different culture seeding densities/splitting ratios. Accordingly, shake flask cultures were seeded from the same starter culture at either low (2.2 × 10^6^ cells/mL, split at 1:9) or high seeding densities (5.8 × 10^6^ cells/mL, split at 1:4). Cultures seeded in the low-density condition experienced a lag phase of two days before entering the exponential growth phase; this lag phase was absent from cultures seeded at the higher cell density of 5.8 × 10^6^ cells/mL (Figure 1D). It was thus determined that a seeding density of approximately 5 × 10^6^ cells/mL and passage at a frequency of 7 days was effective at maintaining healthy cultures and avoiding a prolonged lag phase following subculturing (Figure 1A). Under these passaging conditions, C6/36 suspension cultures exhibited reproducible growth patterns, with an approximate doubling time of 44 h and peak cell densities of up to 2.5 × 10^7^ cells/mL. This high-density seeding regimen was thus adopted for all subsequent experiments, with a 1:4 split of the cultures every 7 days.

**Figure 2:**
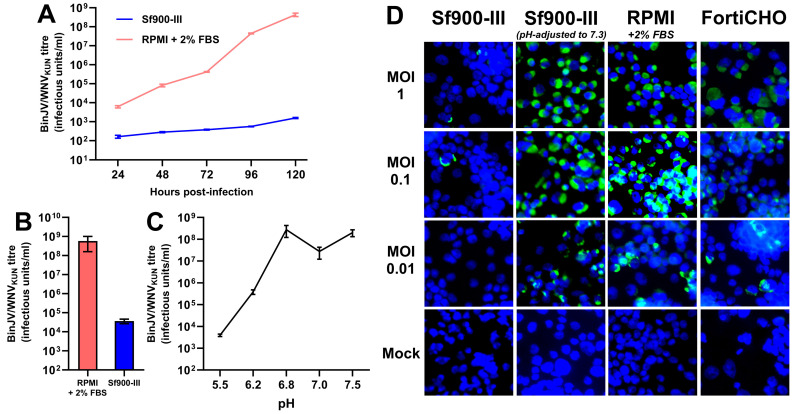
Impact of growth medium and pH on chimeric vaccine candidate BinJV/WNV_KUN_ infectivity and replication. (**A**) BinJV/WNV_KUN_ replication kinetics in adherent T-flask culture supernatant following infection in two different media at MOI of 0.1 as measured by TCID50 ELISA; mean titre of three biological replicates is plotted with error bars representing +/− 1 standard deviation. (**B**) Infectious titre of purified stock of BinJV/WNV_KUN_ in PBS following dilution and titration in two different media. Error bars indicate 95% confidence internal. (**C**) Infectious titre of purified stock of BinJV/WNV_KUN_ following incubation in PBS of various pH values. Error bars indicate 95% confidence internal. (**D**) Immunofluorescence of BinJV/WNV_KUN_ in infected C6/36 monolayers in different media types, based on DAPI (blue)/mAb 4G4 (green) staining of fixed cells at 3 days post-infection, were obtained for BinJV/WNV_KUN_-infected C6/36 cell monolayers in various media types at three different MOIs.

Having demonstrated the successful adaptation of the C6/36 cell line to suspension culture using a serum-free growth medium (Sf900-III), an experiment was performed to first determine if this new serum-free medium could support the replication of insect-specific chimeric orthoflavivirus displaying the antigenic components of West Nile virus (BinJV/WNV_KUN_). This West Nile vaccine candidate was previously shown to replicate to high titres using established protocols whereby C6/36 adherent cultures were grown in RPMI medium supplemented with 2% FBS [20]. To assess the impact of the growth medium on vaccine virus replication, C6/36 cells were seeded in T-flasks directly from a mid-exponential Sf900-III suspension culture, and, following cell attachment, the medium was swapped out with either fresh Sf900-III (serum-free) or RPMI supplemented with 2% FBS. Following the medium swap, adherent cultures were infected with BinJV/WNV_KUN_ at an MOI of 0.1 and the virus titre of the culture supernatant was determined daily for up to five days post-infection (Figure 2A). Whilst BinJV/WNV_KUN_ titres in the serum-supplemented RPMI supernatant increased exponentially over the 5 days, reaching 4.3 × 10^8^ IU/mL by 120 h post-infection, virus titres in Sf900-III increased only linearly and were several orders of magnitude lower at every time point measured, peaking at 1.6 × 10^3^ IU/mL. This difference in virus replication prompted us to further investigate the effect of the two C6/36 growth media on BinJV/WNV_KUN_ infectivity. To perform so, a purified stock of BinJV/WNV_KUN_ was prepared by gradient ultracentrifugation and buffer-exchanged into PBS. Samples of this stock were then subjected to serial dilution and TCID_50_ titration on adherent C6/36 cells grown in 96-well plates, using either Sf900-III or serum-supplemented RPMI as both the dilutant and culture growth medium in either case. A notable decrease in infectious titre was noted when the virus stock was diluted and titrated in Sf900-III, with a drop of over four orders of magnitude compared to the virus diluted and titrated in serum-supplemented RPMI (Figure 2B). This result provided additional evidence that the C6/36 culture growth medium had some influence upon the infectivity of BinJV/WNV_KUN_.

Considering that Sf900-III is a significantly more acidic medium than serum-supplemented RPMI (measured at a pH of 6.2 versus 7.5), it was hypothesised that the pH of the growth medium might influence BinJV/WNV_KUN_ infectivity and/or replication. To investigate this hypothesis, samples of the aforementioned purified virus stock were diluted into a series of pH-adjusted PBS buffers (ranging from 5.5 to 7.5), incubated at room temperature for 5 min, and then immediately titrated on C6/36 cells grown in 96-well plates using serum-supplemented RPMI as both the diluent and culture medium. Reductions in the virus titre of several orders of magnitude were observed when the virus was pre-treated at pH values of 5.5 and 6.2 compared to treatments at higher pH values (Figure 2C). Importantly, the purified BinJV/WNV_KUN_ was shown to lose over 99.8% of its infectious titre when incubated at a pH of 6.2 (the pH of Sf900-III) compared to incubation at a pH of 7.5 (the pH of RPMI). These results indicate that the infectivity of BinJV/WNV _KUN_ was dramatically reduced following exposure to acidic pH, a property previously reported for wild-type orthoflaviviruses including West Nile virus [25,26]. It was thus hypothesised that the acidic pH of Sf900-III was responsible for reducing the infectivity and replication of BinJV/WNV_KUN_ in C6/36 cultures infected in this medium.

To further investigate this lack of viral infectivity in Sf900-III and confirm the effect of medium pH, identical monolayers of C6/36 cells were seeded into six-well plates and, following cell attachment, growth medium was swapped with various different formulations immediately prior to infection with BinJV/WNV_KUN_ at a range of MOIs (1, 0.1, and 0.01). The following four media were tested: (1) Sf900-III (pH: 6.2); (2) Sf900-III adjusted to a pH of 7.3 via the addition of sodium hydroxide; (3) RPMI supplemented with 2% FBS (pH: approximately 7.5); and (4) CD-FortiCHO (a commercially available chemically defined media with a pH of approximately 7.5). It should be noted here that the pH-modified Sf900-III solution underwent precipitation following pH adjustment and this precipitate was removed via filtration prior to utilisation. When the viral antigen (NS1) was visualised via IFA for each culture fixed at 3 days post-infection, the cultures grown in Sf900-III showed only a few infected cells at MOIs of 1 and 0.1, and no infected cells were observed at an MOI of 0.01 (Figure 2D). In contrast, the cultures grown in pH-modified Sf900-III, CD-FortiCHO, and 2% RPMI all exhibited significant levels of infection at each MOI, with a higher number of stained cells observed at higher MOIs (Figure 2D). Together, these results demonstrate that a low culture medium pH (<6.8) was the likely cause of poor BinJV/WNV_KUN_ replication in Sf900-III medium. Although the pH adjustment of this medium to 7.3 appeared to restore virus infectivity, this adjustment was not practical due to high levels of precipitation, reduced cell growth, and an inability to buffer correctly at this pH. Instead, the use of CD-FortiCHO was investigated due to its inherently higher pH and comparable infection results to 2% RPMI in IFA.

Attempts to adapt C6/36 cultures for continuous suspension culture passage in CD-FortiCHO according to the culture parameters used for Sf900-III growth were unsuccessful. It was noted, however, that suspension C6/36 cultures could survive and grow for a transient period when cells grown in Sf900-III were split into CD-FortiCHO at a ratio of 1:4, achieving up to three doublings in cell density (Figure S1). While the initial split into CD-FortiCHO demonstrated a period of proliferation lasting up to six days, this growth phase was transient, as the cells eventually died. Attempts to split these cultures into CD-FortiCHO resulted in complete cell death within 2–3 days (Figure S1), indicating that C6/36 cells could not be maintained as suspension cultures in this medium under the conditions tested. This incompatibility was presumably due to a combination of the medium not being optimised to support the growth of insect cells. Therefore, in order to infect C6/36 suspension cultures with BinJV/WNV_KUN_ and assess its replication, a medium-swap technique was employed whereby C6/36 cells grown in Sf900-III were passaged into CD-FortiCHO at a ratio of 1:4 and immediately infected following a medium swap.

**Figure 3:**
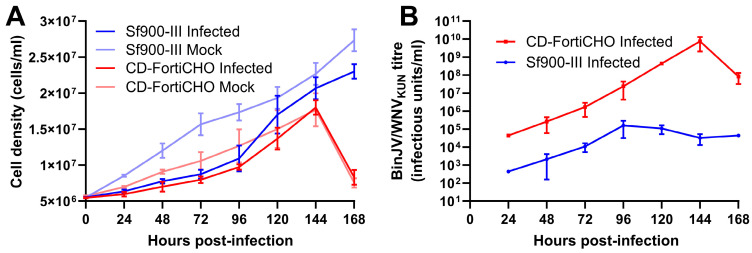
Comparative infection dynamics of C6/36 suspension cultures inoculated with chimeric orthoflavivirus BinJV/WNV_KUN_ (MOI:0.1) in CD-FortiCHO and Sf900-III. (**A**) Suspension culture viable cell density enumerated daily following infection. (**B**) Replication kinetics of BinJV/WNV_KUN_ in suspension culture in two media as approximated by daily TCID50 ELISA on clarified culture supernatant. Each data point plotted is mean of three biological replicates, with error bars representing +/− one standard deviation.

Using this technique, we conducted a direct comparison of BinJV/WNV_KUN_ replication kinetics over seven days in C6/36 cultures in 25 mL shake flasks infected at an MOI of 0.1, in either Sf900-III or CD-FortiCHO. As shown in Figure 3A, all cultures exhibited growth during the experiment; however, the infected cultures proliferated more slowly than the mock-infected cultures for both medium types. Cultures grown in CD-FortiCHO experienced extensive cell death after seven days irrespective of whether or not they were infected, whereas cultures grown in Sf900-III remained healthy and achieved greater cell densities (Figure 3A). However, as shown in Figure 3B, BinJV/WNV_KUN_ titres were at least 100 folds higher in CD-FortiCHO medium compared to Sf900-III medium at all time points tested. In Sf900-III, the peak titre (1.6 × 10^5^ IU/mL) was reached at 96 h post-infection and then began to plateau and decline around this level (Figure 3B). In contrast, virus titres in CD-FortiCHO increased exponentially to a peak titre of 7.6 × 10^9^ IU/mL at 144 h post-infection, exceeding the peak titre observed in Sf900-III cultures by over four orders of magnitude (Figure 3B). Coinciding with the sudden drop in the culture viability of the CD-FortiCHO cultures at 168 h post-infection, the virus titres dropped approximately 100-fold, presumably due to degradation associated with cell lysis.

**Figure 4:**
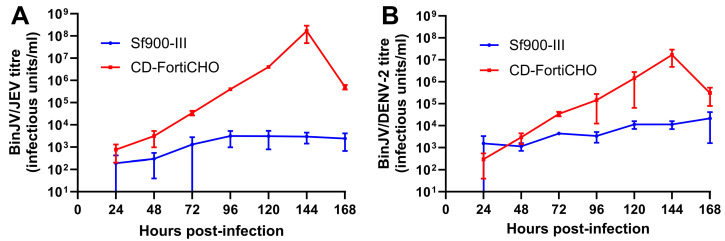
Comparative infection dynamics of C6/36 suspension cultures infected with chimeric orthoflaviviruses BinJV/JEV_NSW22_ or BinJV/DENV2 (MOI:0.1) in CD-FortiCHO and Sf900-III. (**A**) Replication kinetics of BinJV/JEV_NSW22_ in suspension culture in either Sf900-III or CD-FortiCHO as quantified by TCID50 ELISA. (**B**) Replication kinetics of BinJV/DENV-2 in suspension culture in either Sf900-III or CD-FortiCHO as quantified by TCID50 ELISA. Each data point plotted is mean of three biological replicates, with error bars representing +/− one standard deviation.

The medium-swap infection process outlined above was extended to two other chimeric insect-specific orthoflavivirus vaccine candidates: BinJV/JEV_NSW22_, a vaccine candidate for Japanese encephalitis [22], and BinJV/DENV2, a vaccine candidate for dengue fever [27]. Infections were carried out in suspension cultures using parameters identical to those used for BinJV/WNV_KUN_ as above, again comparing Sf900-III and CD-FortiCHO as the culture media. In this set of experiments, for both Sf900-III and CD-FortiCHO, infected and mock-infected C6/36 suspension cultures exhibited broadly similar patterns of cell proliferation (Figure S2) compared to those observed during BinJV/WNV_KUN_ infection (Figure 3A), including the precipitous drop in CD-FortiCHO cultures at the 168 h time point. As shown in Figure 4A, BinJV/JEV_NSW22_ replication kinetics in suspension C6/36 cultures exhibited a similar pattern to that observed during BinJV/WNV_KUN_ infections (Figure 3B), with a peak titre of 1.7 × 10^8^ IU/mL reached at 144 h post-infection in CD-FortiCHO and again dropping significantly at the 168 h time point. Like BinJV/WNV_KUN_, BinJV/JEV_NSW22_ failed to replicate efficiently in Sf900-III, plateauing at 96 h post-infection at titres several orders of magnitude below that in CD-FortiCHO (Figure 4A). BinJV/DENV2 infection kinetics proceeded similarly, although the difference between the two medium types was much less pronounced, and the overall titres in CD-FortiCHO were lower at every time point than those observed for the other two vaccine constructs (Figure 4B). Nevertheless, a similar pattern was observed with BinJV/DENV2 titres in CD-FortiCHO increasing exponentially and peaking at 1.7 × 10^7^ IU/mL at the 144 h post-infection time point, and with titres in Sf900-III plateauing at a low level (Figure 4B).

**Figure 5:**
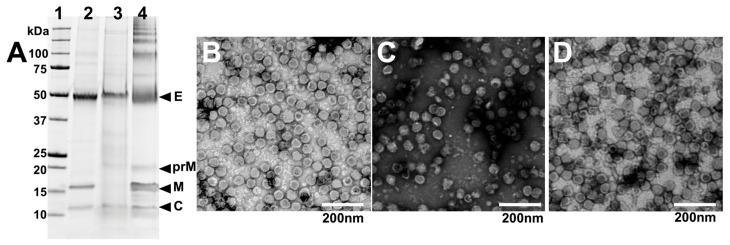
Integrity and purity of virions grown in CD-FortiCHO in suspension culture. (**A**) Purified viruses were analysed using SDS-PAGE. Lane 1 (from left): molecular weight marker ladder (kDa); lane 2: BinJV/WNV_KUN_; lane 3: BinJV/JEV_NSW22_; lane 4: BinJV/DENV2. (**B**) Negative staining transmission electron micrograph of purified BinJV/WNV_KUN_; (**C**) purified BinJV/JEV_NSW22_; (**D**) purified BinJV/DENV2. TEM images are shown at 20,000× magnification and were stained with uranyl acetate.

To further analyse the purity and integrity of virions produced by each of the three chimeric viruses grown in CD-FortiCHO in suspension culture, the triplicate-infected supernatants were harvested and pooled at the conclusion of each experiment (168 h post-infection) and subjected to PEG precipitation followed by gradient ultracentrifugation as per the Materials and Methods Section. After purification, the viruses were analysed by SDS-PAGE. This revealed relatively pure preparations with bands presumed to be the structural proteins, including E (envelope), prM and/or M (pre-membrane or membrane), and C (capsid) proteins being apparently detectable at the expected molecular weights for all three viruses (Figure 5A).

The purified virus preparations were also visualised by negative staining transmission electron microscopy (TEM) to visualise the morphology of the viral particles. This revealed the presence of spherical particles with approximate diameters of 50 nm, consistent with the size and appearance of orthoflavivirus virions (Figure 5B–D), albeit with some degraded particles and debris being present, likely attributed to the harvest time point coinciding with cell culture death. Nonetheless, these data provide evidence that intact particles were produced from C6/36 serum-free suspension cultures.

## 4. Discussion

Previously, we showed that chimeric orthoflaviviruses based on the insect-specific orthoflavivirus BinJV have utility as an emerging platform for producing safe and effective vaccines against orthoflavivirus pathogens [7]. While these chimeric vaccine viruses were shown to replicate to high titres in the C6/36 *Aedes albopictus* mosquito cell line, production has been limited to date to adherent cultures grown in serum-supplemented media, a system that is undesirable for the commercial scale-up and production of vaccines for human use [28]. Furthermore, because the replication of this class of chimeric vaccines is restricted to mosquito cells, traditional cell platforms currently in use for vaccine manufacturing, such as Vero, PER.C6, or Sf9 cells, cannot be leveraged. To overcome these limitations, we determined suitable conditions for culturing C6/36 cells in a serum-free suspension system to facilitate the large-scale production of these chimeric orthoflaviviruses for biotechnology applications. The insights gained here are important for advancing bioprocessing strategies for mosquito cell-based production systems, particularly for emerging chimeric viral platforms.

In this study, we reported a protocol for adapting C6/36 cells to true suspension cultures in commercially available serum-free media (Sf900-III), achieving high peak cell densities (up to 2.5 × 10^7^ cells/mL) with viability consistently above 95%. We determined workable parameters to maintain these suspension cultures (including seeding frequency and seeding density) via dilution into fresh media for a large number of passages (to our knowledge, indefinitely). Maintaining the agitation of the cultures at 250 rpm was crucial for preventing cell aggregation, a major problem observed at lower shaking frequencies, as has been observed by others [16].

The successful cryopreservation and revival of the suspension-adapted C6/36 cells in Sf900-III further demonstrated that this protocol provides a stable foundation for research and biotechnology applications including the production of master and working cell banks. While C6/36 cells exhibited robust and reproducible growth in suspension culture in Sf900-III, when we attempted to grow a chimeric orthoflavivirus vaccine virus (BinJV/WNV_KUN_) in this culture system, viral titres were several orders of magnitude lower than that achieved with a standard medium (serum-supplemented RPMI) in adherent cultures. Further analysis of the media indicated that the slightly acidic pH of SF900III (6.2) versus the slightly alkaline pH of RPMI (7.5) was likely responsible for the low yields of BinJV/WNV_KUN_ in the former. We concluded that the infectivity of the virus was greatly reduced following brief exposure to a pH of less than 6.8. These findings align with earlier studies [29,30], which have highlighted the loss of infectivity for WNV under acidic conditions. This observed loss in infectivity is attributed to irreversible conformational changes in orthoflavivirus particles exposed to an acidic environment that mirror alterations in the E protein typically triggered within acidic endosomes during the natural course of viral entry into host cells [25]. While this is a critical step for orthoflaviviruses to facilitate membrane fusion and release their genetic material into the host cell cytoplasm, in an extracellular environment, this renders virus particles non-infectious [25,26]. Sf900-III is formulated to uphold a relatively constant and controlled pH of 6.2, buffered by phosphate [31]. When we chemically raised the pH of Sf900-III by the addition of sodium hydroxide, a substantial precipitate formation was observed that impaired its ability to support cell growth and buffer effectively at an elevated pH, an observation that was previously reported by others [31] when attempting to raise the pH of highly similar Sf900-II medium to support Sf9 cell growth. Consequently, it was determined that Sf900-III medium is not appropriate for orthoflavivirus cultures.

We also investigated the use of an alternate serum-free and chemically defined medium, CD-FortiCHO, to grow the chimeric orthoflavivirus vaccine viruses. This commercially available medium has a slightly alkaline pH (7.5) and was specifically developed for use in pharmaceutical industry for the production of therapeutic biologics in Chinese hamster ovary cultures. However, when assessed as a medium for culturing C6/36 cells in suspension, CD-FortiCHO could not support their sustained growth, apparently lacking some essential growth component required to maintain the suspension cells at a high density or else being incompatible with the culturing parameters tested herein. Nevertheless, by using an initial 1:4 split of a suspension culture of C6/36 cells grown in Sf900-III in CD-FortiCHO, we were able to grow cells for a limited time in suspension sufficient to facilitate infection and virus replication. Furthermore, this medium-swap approach allowed the growth of BinJV/WNV_KUN_ to peak titres exceeding those observed with traditional serum-supplemented RPMI medium in adherent cultures.

A major objective of this study was to apply a C6/36 suspension culture system to grow high yields of chimeric orthoflaviviruses for an enhanced bioprocess for vaccine production. In addition to BinJV/WNV_KUN_, this also included BinJV/DENV2 [27] and BinJV/JEV_NSW22_ [22]. Like BinJV/WNV_KUN_, we also observed a notable reduction in virus replication for BinJV/JEV_NSW22_ and BinJV/DENV2 when cultured in Sf900-III. This is consistent with the negative effect of acidic pH on orthoflavivirus infectivity as discussed above and confirms the lack of suitability of this medium for chimeric orthoflavivirus production in general. Similarly to BinJV/WNV_KUN_, a media swap into CD-FortiCHO restored the replication of BinJV/JEV_NSW22_ and BinJV/DENV2 to high titres, providing a proof of concept that these chimeric vaccines viruses can be cultured in a serum-free medium suspension culture system. We also demonstrated that the integrity of the chimeric orthoflaviviruses remained preserved after purification from infected suspension C6/36 cultures, with TEM analysis showing typical spherical particles, approximately 50 nm in size. Overall, these results indicate that the bioprocess developed can work across multiple orthoflavivirus vaccine candidates based on the chimeric, insect-specific BinJV platform [17], paving the way for the future development of an efficient and scalable serum-free vaccine manufacturing bioprocess using the C6/36 cell line.

This extension of the suspension culture system to multiple BinJV chimeric vaccine candidates demonstrates the utility of suspension-adapted C6/36 cell lines for the production of a variety of vaccine candidates. There may be scope to expand these findings further by testing the utility of this C6/36 suspension culture system to replicate arbovirus vaccines based on other vaccine technologies, potentially delivering superior yields. These opportunities include the replication of wild-type arboviruses (for inactivation), live-attenuated arbovirus vaccines, and other chimeric systems such as those based on the insect-specific Eilat virus (EILV) [32] or Yada Yada virus (YYV) [33] alphavirus backbones. The suspension culture of C6/36 cells may also have other uses in biotechnology beyond the production of arbovirus vaccines, such as the production of recombinant proteins using the non-lytic BacMos system [34]; indeed, one such application for C6/36 suspension cultures was previously demonstrated for the production of densoviruses as mosquito biological control agents [16,35].

One limitation of the present study was the lack of the provenance of the C6/36 line used in this study, with initial cultures derived from laboratory stocks of unknown passage and with passage records of the line not being available. To confirm the identity of the cell line, we PCR-amplified and Sanger-sequenced a portion of the Dicer-2 gene from DNA extracted from our stock of cells(Figure S3); the recovered sequence matched that published for the reference genome of the C6/36 cell line [36]. However, we cannot be certain that the suspension culturing and virus infections processes developed here will proceed in the same way in other C6/36 lineages. We are currently in the progress of repeating these preliminary findings with a well-defined C6/36 cell bank of known provenance obtained from the American Type Culture Collection (ATCC) repository (ATCC CRL:1660) [5].

Another major limitation relates to the culturing parameters used for suspension studies and their interaction with the buffering systems used in the various medium types to maintain pH. Insect cell serum-free growth media utilise a phosphate-buffering system designed to maintain a low pH environment of 6.2–6.4. This system does not require carbon dioxide to buffer and, thus, insect cultures are grown at ambient carbon dioxide levels [37]. In this study, due to equipment limitations, all suspension cultures and infections took place in vented Erlenmeyer flasks in an incubator at ambient CO_2_. This environmental influence may have contributed to the abrupt cell death observed in cultures grown in CD-FortiCHO, which utilises a bicarbonate-based buffering system. Bicarbonate-buffered media rely on the presence of carbon dioxide to maintain an appropriate pH range of 7–8; carbon dioxide helps stabilise the pH by interacting with the bicarbonate ions in the medium [38], preventing the cultures from becoming too alkaline. In this study, we relied on a medium-swap technique to facilitate virus production, growing the seed stock of C6/36 cells in Sf900-III and then swapping to the more alkline CD-FortiCHO at the time of infection. While this proof-of-concept study demonstrated robust virus replication, it is not clear that such a technique is practical for a large-scale bioprocess, where a single medium capable of supporting culture growth and virus production may be preferable. Overall, these observations highlight the importance of developing a bespoke serum-free insect cell medium capable of being buffered in a more neutral/alkaline pH range (7–8) necessary to replicate non-baculovirus viral constructs.

## 5. Conclusions

In conclusion, the findings of this study expanded upon prior investigations into C6/36 serum-free suspension cultures for virus production by refining and documenting protocols for adaption to serum-free suspension cultures and achieving the reliable cryopreservation and revival of the cells in this system. We also developed a strategy to culture orthoflaviviruses in C6/36 suspension cultures, by transitioning into a chemically defined medium to provide high virus yields. These outcomes represent a significant advance in developing C6/36 culture systems for scaled-up bioprocesses for vaccine manufacture, serving as a robust starting point for the future assessment of production in instrumented bioreactors. The ability to efficiently produce orthoflavivirus vaccines in suspension cultures of the C6/36 mosquito cell line using CD-FortiCHO demonstrates the system’s potential for high-yield and scalable production and highlights the requirement for an appropriate pH in the medium for the optimal yield of these viruses. This finding suggests that the C6/36 cell line shows promise as a major industrial insect cell line for arbovirus vaccine production. The adaptability and robustness of the C6/36 cells in these optimised conditions could streamline the production process, reduce costs, and improve the availability of vaccines against arboviruses such as DENV, WNV, and JEV. This advancement not only addresses the need for more efficient vaccine production methods but also enhances public health efforts by providing a reliable and scalable platform for producing critical vaccines, ultimately combating these significant global health threats more effectively.

## Data Availability

Data supporting the findings of this study are included within the article. Additional data can be obtained from the corresponding author upon reasonable request.

## Supplementary Materials

The following supporting information can be downloaded at https://www.mdpi.com/article/10.3390/v17020250/s1. Figure S1. C6/36 suspension culture growth kinetics in two medias demonstrating behaviour of cultures following medium-swap from Sf900-III to CD-FortiCHO (CDM). Suspension cultures grown in Sf900-III were periodically counted during growth and passaging. Culture behaviour following a medium swap (1:4) dilution from Sf900-III to CD-FortiCHO was monitored, including an attempted passage of the swapped culture into 100% CD-FortiCHO, resulting in culture death. Figure S2. C6/36 suspension culture growth kinetics following infection with chimeric flaviviruses;(A): Cell density over time of the suspension culture infected with BinJV/JEV vs non-infected cells grown in SF900-III media. (B): Cell density over time of the suspension culture infected with BinJV/JEV vs non-infected cells grown in CD-FortiCHO media (CDM). (C): Cell density over time of the suspension culture infected with BinJV/DENV-2 vs non-infected cells grown in SF900-III media. (D): Cell density over time of the suspension culture infected with BinJV/DENV-2 vs non-infected cells grown in CD-FortiCHO media. Each data point plotted is the mean of three biological replicates, with error bars representing +/- one standard deviation. Figure S3. Sequence confirmation of the identity of the C6/36 cell line used in this study; (A): Agarose gel image showing amplification of Dicer-2 gene of C6/36 cells by PCR. Lane 1: Positive control – amplicon generated from Sf9 gDNA template with primers specific for the Sf9 DICER-2 region. Lane 2: Amplicon derived from C6/36 gDNA cells using Aedes albopictus DICER-2-specific primers. Lane 3: No template control. Lane 4: 1kb DNA ladder. (B): Sanger sequencing reads of the Dicer-2 gene PCR-amplified from genomic DNA extracted from the C6/36 cell line used in this study. Alignment (containing forward and reverse reads (2_R_E03 and 2_F_E03) around the site of Dicer-2’s 1-bp deletion) for the C6/36 Dicer2 gene fragment. The reads are aligned to the Aedes albopictus reference genome (A. albopictus Dicer 2 isoform A (Dcr-2)) and the published C6/36 cell line genome (A. albopictus cell-line C6/36 Dicer 2 (Dcr-2). Deleted nucleotide highlighted by red oval.
