# Supplementary material for: Serum-Free Suspension Culture of the Aedes albopictus C6/36 Cell Line for Chimeric Orthoflavivirus Vaccine Production
Source: Viruses. 2025 Feb 12;17(2):250. doi: 10.3390/v17020250 (PMC11860912; doi:10.3390/v17020250)
Supplement: Supplementary file 1 [file viruses-17-00250-s001.zip › viruses-3388498-supplementary.pdf]

SUPPLEMENTARY INFORMATION

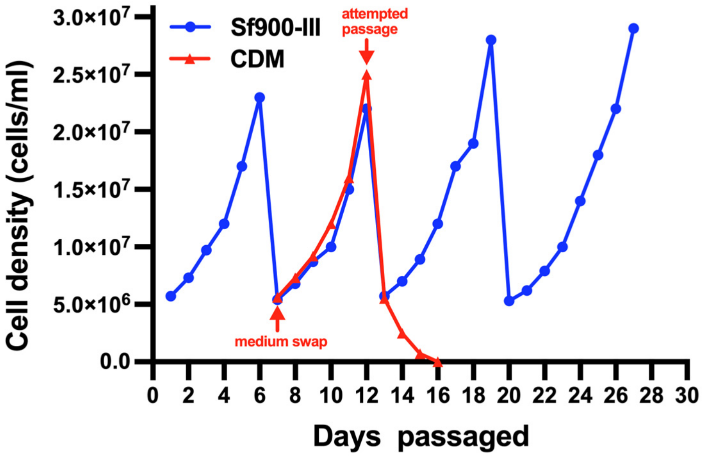

Figure S1. C6/36 suspension culture growth kinetics in two medias demonstrating behaviour of cultures following medium-swap from Sf900-III to CD-FortiCHO (CDM). Suspension cultures grown in Sf900-III were periodically counted during growth and passaging. Culture behaviour following a medium swap (1:4) dilution from Sf900-III to CD-FortiCHO was monitored, including an attempted passage of the swapped culture into 100% CD-FortiCHO, resulting in culture death.

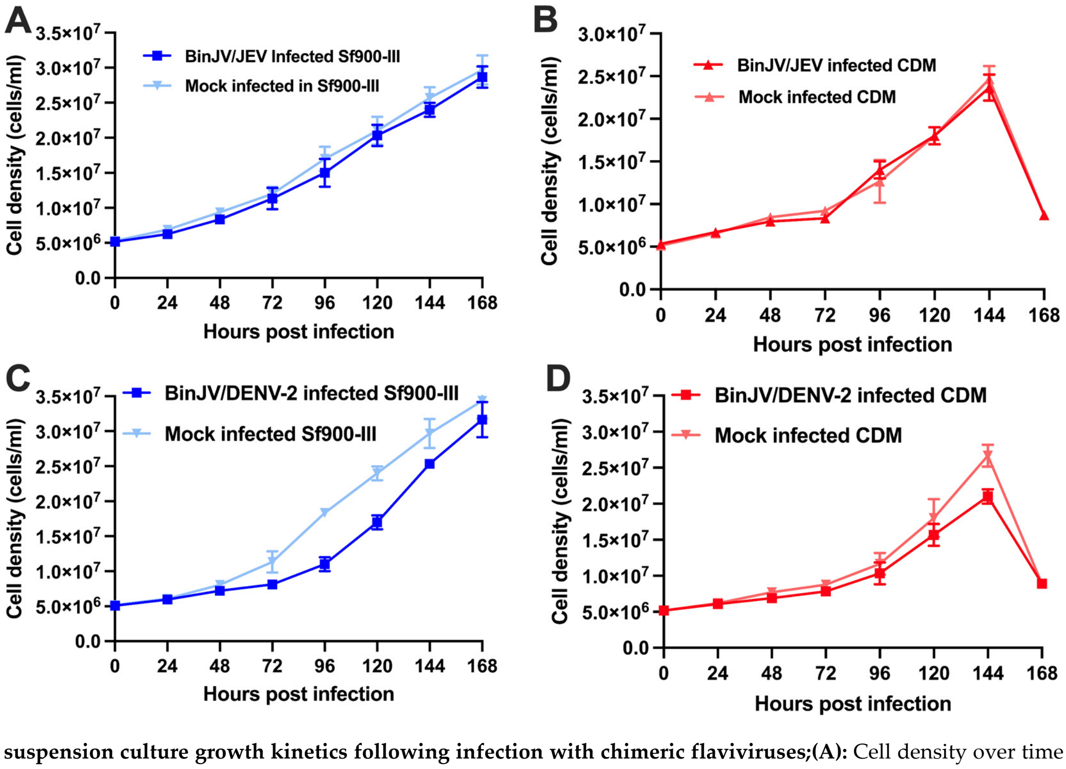

Figure S2. C6/36 suspension culture growth kinetics following infection with chimeric flaviviruses;(A): Cell density over time of the suspension culture infected with BinJV/JEV vs non-infected cells grown in SF900-III media. **(B):** Cell density over time of the suspension culture infected with BinJV/JEV vs non-infected cells grown in CD-FortiCHO media (CDM). **(C):** Cell density over time of the suspension culture infected with BinJV/DENV-2 vs non-infected cells grown in SF900-III media. **(D):** Cell density over time of the suspension culture infected with BinJV/DENV-2 vs non-infected cells grown in CD-FortiCHO media. Each data point plotted is the mean of three biological replicates, with error bars representing +/- one standard deviation.

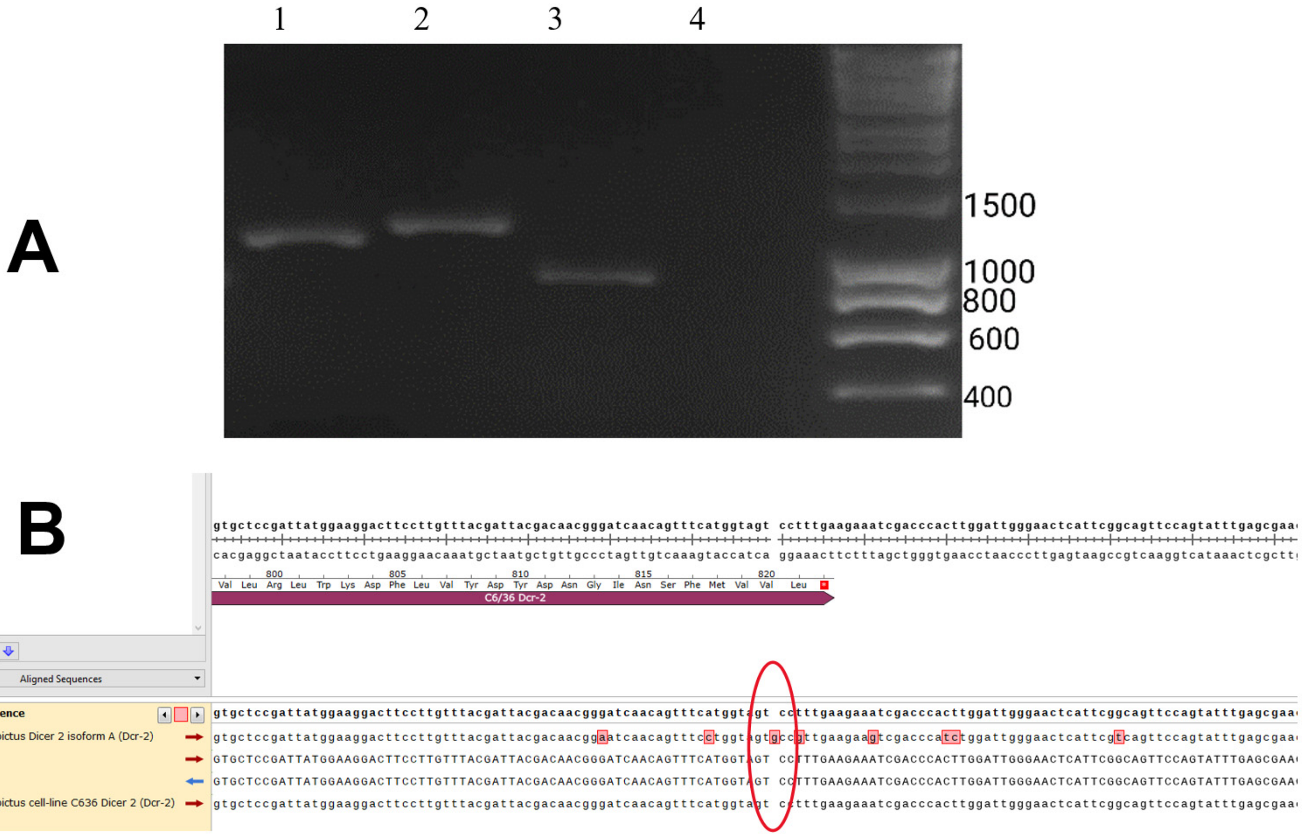

Figure S3. Sequence confirmation of the identity of the C6/36 cell line used in this study; (**A**): Agarose gel image showing amplification of Dicer-2 gene of C6/36 cells by PCR. Lane 1: Positive control – amplicon generated from Sf9 gDNA template with primers specific for the Sf9 DICER-2 region. Lane 2: Amplicon derived from C6/36 gDNA cells using Aedes albopictus DICER-2-specific primers. Lane 3: No template control. Lane 4: 1kb DNA ladder. (**B**)**:** Sanger sequencing reads of the Dicer-2 gene PCR-amplified from genomic DNA extracted from the C6/36 cell line used in this study. Alignment (containing forward and reverse reads (2_R_E03 and 2_F_E03) around the site of Dicer-2's 1-bp deletion) for the C6/36 Dicer2 gene fragment. The reads are aligned to the *Aedes albopictus* reference genome (*A. albopictus* Dicer 2 isoform A (Dcr-2)) and the published C6/36 cell line genome (*A. albopictus* cell-line C6/36 Dicer 2 (Dcr-2). Deleted nucleotide highlighted by red oval.
